# Globalizing newborn screening: bridging gaps in genetic diagnosis and treatment

**DOI:** 10.3389/fped.2025.1672993

**Published:** 2025-10-16

**Authors:** Huma Tariq, Vincenzo Salpietro, Henry Houlden, Giovanni Stevanin

**Affiliations:** ^1^ Health Biotechnology Division, National Institute for Biotechnology and Genetic Engineering (NIBGE), Faisalabad, Pakistan; ^2^ Pakistan Institute of Engineering and Applied Sciences (PIEAS), Islamabad, Pakistan; ^3^ European Brain Research Institute, Rome, Italy; ^4^ Department of Neuromuscular Disease, UCL Queen Square Institute of Neurology, London, United Kingdom; ^5^ CNRS UMR5287, INCIA, EPHE, University of Bordeaux, Bordeaux, France

**Keywords:** globalizing NBS, treatable disorders, genome-based NBS, gene panels, LMIC (low and middle income countries)

A comprehensive perspective on the evolving landscape of newborn screening (NBS) has recently highlighted its increasing complexity and transformative potential in the genomic era (1). The fundamental aim of NBS is to detect conditions early enough to enable timely interventions before irreversible damage occurs. Traditional NBS relies on biochemical markers to screen for conditions such as phenylketonuria (PKU) and congenital hypothyroidism, which, when detected early, can be managed with dietary or hormonal therapies that prevent cognitive impairment and growth delay. NBS for treatable, early-onset disorders is recognized as a crucial public health measure at the international level and it provides multiple benefits, including early treatment guidance, genetic counseling, and psychosocial assistance to the patient and close family. However, when dealing with gene panel based NBS, inclusion of a new condition into NBS gene panels remains a slow and complex process, which takes many years; on average 9.5 years in a high-resource settings like the United States (US). This delay means that some clinically actionable genetic disorders remain undetected or are introduced too late into screening protocols, potentially missing the critical window for intervention.

It is important to distinguish between early diagnosis and newborn screening. Early diagnosis refers to testing symptomatic or high-risk children to confirm disease, while newborn screening is a population-based, presymptomatic tool designed to detect treatable disorders at birth. Although distinct, these approaches are complementary: the same genomic strategy applied at birth functions as NBS, and when applied later in symptomatic infants, it serves as a diagnostic tool. Both pathways generate valuable genetic information that can feed into the optimization of NBS panels and improve long-term patient care. WHO also cautions that while newborn screening can identify potential risks, it should not be misrepresented as a definitive diagnostic tool—follow-up confirmatory testing remains essential to ensure accuracy and avoid misinterpretation.

We wish to make some additional comments and suggestions regarding the implementation of NBS in developing countries, where the same screening strategies are not applied, particularly in underserved ethnic groups and economically compromised/disadvantaged regions. Current NBS gene panels may not reflect the true burden of disease in these populations, which often have distinct genetic architectures. Ideally, the selection of disorders for genetic NBS panel should be informed by the most prevalent genes and disease-causing variants in each geographical and ethnic context.

While some countries have implemented government-supported, nationwide NBS programs, others limit those services to high-risk populations. Unfortunately, NBS is not yet implemented in many developing countries although these countries harbor more than half of the total global births. For instance, sickle cell disease—accounting for nearly 75% of the global burden in sub-Saharan Africa and with significant prevalence in South Asia—has well-established, inexpensive NBS protocols that could be scaled up as an entry point for broader screening. However, proper global efforts are required to ensure that such initiatives move beyond regional boundaries and address infants as a shared global responsibility (2). Due to this gap, timely diagnosis and adequate treatment pose a major challenge, contributing to higher neonatal mortality and related health complications. Data obtained from some developing countries indicate a higher-than-anticipated prevalence of inherited metabolic diseases, many of which are diagnosed too late to prevent serious complications (3). Expanding NBS as a coordinated global public health strategy would not only improve early detection and outcomes but also yield more accurate prevalence data across diverse populations. Such data could guide the development of more inclusive and effective screening panels that reflect regional genetic diversity (4). Currently, 1 in every 294 newborns is identified through NBS programs in USA to have a manageable condition, often allowing early treatment and lifesaving interventions as well as long-term quality of life improvements. This number would likely be increased if more actionable genetic disorders, such as spinal muscular atrophy (SMA, early diagnosis enables administration of gene therapy) or biotinidase deficiency (treatable with biotin supplementation) were included in screening targets (5).

Unfortunately, our understanding of the genetic basis of many pediatric disorders remains incomplete, especially in developing countries. This limited understanding largely stems from underutilization of genetic methods, itself a consequence of restricted access to genomic technologies, research infrastructure, and population-specific genomic data. Diagnostic efficiency is further compromised by the lack of incorporation of disease characterization data from areas with high rates of consanguineous marriages, where autosomal recessive disorders are more prevalent. Critically, these regions remain underrepresented in genetic research, resulting in insufficient data to inform NBS panel design. This underrepresentation highlights the urgent need for *pan-population genomics* to capture diversity across low- and middle-income countries (LMICs). Without the inclusion of these populations, genomic NBS risks reinforcing existing inequities rather than addressing them. More comprehensive and representative data are essential to develop optimal test panels for NBS that reflect true global disease prevalence, rather than relying primarily on data from high-income countries.

Recent technological advancement has established genome sequencing (GS) as a promising tool for NBS in the near future. Whole genome and exome sequencing have demonstrated their ability to detect both common and rare genetic conditions, many of which are not currently captured by biochemical screening methods or clinical evaluation. To fully realize the potential of genome-based NBS, it is essential to promote global inclusivity in genomic research. Random sampling, families' recruitment and genomic analysis of patients from all around the globe irrespective of their region, religion, ethnicity, and economic background will generate a more complete understanding of disease causes to be employed for more equitable and effective applicable NBS programs worldwide.

Resistance to genome-based NBS, often due to concerns about cost, incidental findings and data interpretation, can be managed by emphasizing its benefits over current methods. Beyond cost, ethical and privacy considerations remain critical. WHO and other international bodies emphasize the need to address challenges such as informed consent, responsible return of results, long-term storage and security of genomic data, and questions of data ownership. These issues are particularly important in low- and middle-income countries (LMICs) and must be explicitly integrated into policy and implementation frameworks before large-scale adoption of genomic newborn screening can be achieved. Although the current cost of GS is approximately USD 300–500 per sample, sequencing costs continue to decline. Moreover, strategic collaborations with developed countries could facilitate implementation in resource-limited settings, while the generated data will enable the design of cost-effective, population-specific panels for the future. Such partnerships could also involve technology transfer, shared cloud-computing infrastructure for data analysis, and training programs for local clinicians and scientists.

The benefits of genome-based NBS can be further enhanced by well-organized, symptom-driven, and ethnically guided genetic screening programs. An effective initial approach may begin with the organized recruitment of patients based on clinical symptoms and detailed phenotyping (evaluated by expert clinicians) and family history. Targeted genetic screening of these patients may yield more direct and actionable information than untargeted mass screening. On the contrary, untargeted screening such as WES or GS has enabled the identification of unexpected observations, including multiple inheritance modes associated with several causative genes (SPG4, REEP2) (6, 7) and multiple diseases associated with a given gene (PNPLA6) (8). Such targeted and untargeted screenings are generating a large volume of clinically relevant data, and their extension to the worldwide population will better support the design of population-specific, next-generation NBS panels. Together, these complementary approaches—targeted for immediate diagnosis and untargeted for broader discovery—offer the most effective pathway to optimize future newborn screening programs. Importantly, this collaborative data generation is not only essential for building globally representative screening panels but also empowers low- and middle-income countries (LMICs) by fostering local genomic research infrastructure, developing skilled human resources, and strengthening long-term capacity for precision medicine.

In alignment with global health priorities, the World Health Organization (WHO) has recently advocated for universal newborn screening for several critical health conditions such as bilateral hearing loss, eye abnormalities and hyperbilirubinemia as part of essential postnatal care emphasizing that every newborn, regardless of geographic or economic background, deserves access to early detection and intervention for treatable conditions. We strongly recommend this should be extended to all genetic and metabolic health issues for which patients may be treatable or at least can benefit from early diagnosis and proactive management.

In support to this approach, a recent study evaluated the utility of genomic sequencing vs. a targeted neonatal gene-sequencing panel for suspected genetic disorders. Among 400 newborns who underwent both tests simultaneously, genome sequencing showed a better diagnostic rate of 49% as compared to the targeted panel sequencing, 27% (containing 1,722 genes implicated in early-onset disorders). Remarkably, 164 pathogenic variants missed by the gene panel were identified through GS, highlighting the better sensitivity and broader diagnostic reach of genome-wide approaches (9). This finding demonstrates that even well-designed and comprehensive panels from developed countries may fail to detect causative mutations, particularly in genetically diverse or underrepresented populations.

In broader perspective, GS based newborn screening programs should be designed as global collaborations rather than isolated national efforts. It is time to establish international consortia dedicated to each genetic condition, so that newborns worldwide can access ideal screening for treatable disorders, using the Ataxia-Global initiative as a model (https://ataxia-global-initiative.net/). Such collaborative frameworks would not only enable the pooling of clinical and genomic data across diverse populations but also accelerate the development of more inclusive, evidence-based screening protocols. This approach is not only a service to humanity but also a strategic investment to improve worldwide health infrastructure. Importantly, in many developing countries, advanced therapies such as gene therapy and other sophisticated molecular interventions may remain prohibitively expensive and out of reach for the foreseeable future. In these settings, early detection through NBS can offer a critical window for prevention, family planning, and timely clinical management—often the most realistic and impactful intervention available. Moreover, valuable lessons can be drawn from successful screening models in genetically isolated populations, such as Ashkenazi Jewish and Finnish communities, where targeted screening for founder mutations has led to effective carrier detection and disease prevention. A similar approach could be applied in certain developing countries, particularly those with high rates of consanguinity and a correspondingly elevated prevalence of autosomal recessive disorders. By identifying region-specific founder mutations, targeted and cost-effective screening strategies could be developed, improving diagnostic yield and public health impact.

By uniting global efforts, resources and prioritizing inclusivity, we can significantly reduce the risk of missed diagnoses and bring equitable, actionable newborn screening to all regions of the world ultimately improving global health outcomes.
